# Work interruptions and nurse turnover intention mediated by burnout

**DOI:** 10.3389/fpubh.2026.1838436

**Published:** 2026-06-24

**Authors:** Cong Wang, Hongfang Zhu

**Affiliations:** Department of Nursing, Sir Run Run Shaw Hospital, School of Medicine, Graduate School, Zhejiang University, Hangzhou, China

**Keywords:** burnout, conservation of resources, coping resources, nursing work interruptions, occupational health, psychosocial work factors, serial mediation, turnover intention

## Abstract

**Introduction:**

Nursing work interruptions represent common psychosocial workplace exposures that may contribute to burnout and threaten workforce stability.

**Methods:**

A cross-sectional online survey was conducted among nurses (*N* = 261) working in general inpatient wards within a four-campus tertiary hospital system in Zhejiang Province, China, from 14 January to 10 February 2026. Interruptions were assessed using the Nursing Work Interruption Scale (NWIS; total score), coping resources were measured using a 26-item interruption coping resources questionnaire (total score), burnout was evaluated using the Copenhagen Burnout Inventory (CBI; 0–100 converted mean score), and turnover intention was measured using a 3-item scale (mean score). Mediation and serial mediation analyses were performed using PROCESS version 5.0 with 5,000 bias-corrected bootstrap samples.

**Results:**

Turnover intention demonstrated a significant positive association with interruptions (*c* = 0.015, *p* = 0.006). Burnout mediated this relationship [Model 4 indirect = 0.0155, 95% BC CI (0.0104, 0.0216); *c*′ = −0.0006, *p* = 0.917]. In the serial mediation model (Model 6), interruptions were associated with lower coping resources (*b* = −0.3210, *p* < 0.001) and higher burnout (*b* = 0.5704, *p* < 0.001); coping resources were associated with lower burnout (*b* = −0.7034, *p* < 0.001); and burnout was associated with higher turnover intention (*b* = 0.0178, *p* < 0.001). Indirect effects through burnout [0.0102, 95% BC CI (0.0063, 0.0155)] and serially through coping resources and burnout [0.0040, 95% BC CI (0.0019, 0.0077)] were found to be significant. These results remained robust after adjusting for variables such as department group, years working in the hospital, and average daily patient volume [Model 6 Ind2 = 0.0100, 95% BC CI (0.0061, 0.0155); and Ind3 = 0.0035, 95% BC CI (0.0015, 0.0069)].

**Discussion:**

The findings are consistent with a conservation of resources (COR)-informed resource depletion pathway, linking interruptions to turnover intention primarily through burnout. This highlights a potential modifiable occupational health target for prevention.

## Introduction

Nurse retention is increasingly recognized as a significant issue for workforce sustainability and occupational health (1, 2). In inpatient settings, nurses often experience time pressure, the need to multitask, and high cognitive demands. In these contexts, work interruptions are common and have been defined as events that temporarily suspend a primary task, necessitating a shift in attention (3). These interruptions may be conceptualized as psychosocial workplace hazards.

Interruptions may also be conceptualized based on the conservation of resources (COR) theory (4, 5), which suggests that interruptions pose ongoing challenges to valued resources such as time, attention, emotional energy, perceived control, and social support from coworkers (6–8). Repeated interruptions may deplete nurses’ coping resources, intensify burnout, and increase withdrawal-related cognitions such as turnover intention, thereby jeopardizing workforce stability (9, 10).

Recent international studies have continued to highlight the relevance of occupational stress, resilience, burnout, and turnover-related outcomes among nurses during the pandemic, post-pandemic, and high-pressure healthcare contexts. Studies involving intensive care nurses during the Coronavirus disease 2019 (COVID-19) pandemic have reported high stress and low resilience levels (11), while post-pandemic evidence suggests that moral resilience is associated with lower levels of burnout, quiet quitting, and turnover intention among nurses (12). Recent mediation research has also linked work engagement, resilience, and turnover intention (13), and network analyses have further highlighted the relationship between burnout and turnover intention among nurses (14). However, less is known about how routine ward-level interruptions, such as everyday psychosocial work exposures, are associated with coping resources, burnout, and turnover intention among general inpatient ward nurses.

### Aim and hypotheses

This study examined whether burnout mediates the relationship between nursing work interruptions and turnover intention, and whether coping resources and burnout sequentially mediate this association.

H1: Interruptions are positively associated with turnover intention.

H2: Interruptions are negatively associated with coping resources.

H3: Interruptions are positively associated with burnout.

H4: Coping resources are negatively associated with burnout.

H5: Burnout mediates the relationship between interruptions and turnover intention, with a serial indirect effect (interruptions → coping resources → burnout → turnover intention).

## Materials and methods

### Design, setting, and participants

This cross-sectional online survey was conducted among nurses working in general inpatient wards within a four-campus tertiary hospital system in Zhejiang Province, China, from 14 January to 10 February 2026. The four campuses belonged to the same hospital system and shared a unified nursing management structure, similar organizational policies, and broadly comparable ward workflows. A convenience sampling method was employed for this study. The questionnaire link was distributed via a QR code to the head nurses of 32 participating general inpatient wards, each with approximately 15–20 nurses. The head nurses were instructed to forward the survey invitation to eligible nurses within their wards. Emergency departments, intensive care units, operating rooms, and other non-general ward units were excluded from the survey. Since the researchers could not verify the exact number of nurses who received, viewed, or opened the QR code after it was forwarded, the denominator required to calculate a formal response rate was unavailable. Therefore, a formal response rate could not be calculated. A total of 261 completed questionnaires were included in the final analysis.

### Measures

Nursing work interruptions were measured using the 12-item Chinese version of the Nursing Work Interruption Scale (NWIS), originally developed by Yu and Lee (15) (comprising two domains: human and environmental factors), and translated and psychometrically validated by Chen et al. (16). Items were rated based on a 6-point frequency scale (1 = almost never; 2 = 1–2 times/week; 3 = 3–4 times/week; 4 = 1–2 times/day; 5 = 3–4 times/day; and 6 = ≥5 times/day). Higher total scores indicated more frequent interruptions.

Coping resources (*M*1) were assessed using the 26-item interruption coping resources questionnaire [De (17)], with items rated on a scale from 1 (strongly disagree) to 5 (strongly agree). The total score was calculated based on these ratings.

Burnout (*M*2) was measured using the 19-item Copenhagen Burnout Inventory (CBI), originally developed by Kristensen et al. (18) and translated into Chinese by Fong et al. (19). Responses were converted to a standardized metric (such as 0, 25, 50, 75, and 100), and the mean burnout score was computed, with higher scores indicating greater burnout.

Turnover intention (*Y*) was measured using a 3-item scale based on Mobley et al. (20), and this was adapted and culturally validated by Yen (21) for China. The items were measured using a 5-point Likert scale, and the average was computed with the result ranging from 1 to 5. The higher the result, the higher the turnover intention.

### Ethics consideration

Ethical approval was obtained from the Ethics Committee of Sir Run Run Shaw Hospital, Zhejiang University School of Medicine (Approval No. 2025–2024, Acceptance No. 2025–4,080-01; Date: 23 December 2025). This study adhered to the principles outlined in the Declaration of Helsinki. Before accessing the questionnaire, participants were presented with an electronic informed consent statement explaining the study purpose, voluntary nature of participation, anonymity, confidentiality, and the right to decline participation. Participants who did not agree did not proceed to the questionnaire. Continuing to complete the anonymous online questionnaire was regarded as providing electronic informed consent. No personal identifying information was collected.

### Sample size justification

A formal *a priori* sample size calculation was not conducted before recruitment. For transparency, sample size adequacy was assessed based on the largest covariate-adjusted regression model used in the PROCESS analyses. The largest model included seven predictors: nursing work interruptions, coping resources, burnout, two dummy variables for department group, years of working in the hospital, and average daily patient volume. Assuming a medium effect size (*f*^2^ = 0.15), *α* = 0.05, and a statistical power of 80%, the minimum required sample size was approximately 103 participants. The final analytic sample of 261 nurses exceeded this requirement and provided more than 30 participants per predictor. In addition, indirect effects were estimated using 5,000 bias-corrected bootstrap samples.

### Common method variance

Since the main study variables were collected using self-reported questionnaires at a single time point, common method variance may be a concern. Several procedural steps were used to reduce this risk. The survey was administered anonymously, no personal identifying information was collected, participants were informed that their responses would be used only for research purposes, and standardized instructions were provided before the completion of questionnaire. In addition, different validated scales were used to measure interruptions in nursing work, coping resources, burnout, and turnover intention. Nevertheless, common method variance cannot be completely ruled out and is acknowledged as a limitation of this study.

### Statistical analysis

Analyses were performed using SPSS 27.0 and PROCESS v5.0 (22). Descriptive statistics and Cronbach’s alpha values were computed. The Pearson correlation analyses (two-tailed) examined relationships among core variables. To describe the types of interruptions experienced by nurses, item-level means and standard deviations for the 12 NWIS items were calculated. One-way analysis of variance (ANOVA) was performed to compare total NWIS scores across department groups and average daily patient volume categories, with the Welch’s test and Games–Howell *post-hoc* comparisons applied as robustness checks. Mediation (Model 4) and serial mediation (Model 6) analyses were conducted using 5,000 bias-corrected bootstrap samples with 95% confidence intervals (95% CI). Adjusted models included the department group, years of working in the hospital, and average daily patient volume as covariates. The department group was entered using two dummy variables, with internal medicine wards as the reference group.

## Results

### Participant characteristics

Participant characteristics are presented in Table 1.

**Table 1:** Participant characteristics (*N* = 261).

Characteristic	Category	*n* (%)
Sex	Female individuals	261 (100.0)
Age (years)	20–25	91 (34.9)
	26–30	54 (20.7)
	31–35	60 (23.0)
	36–40	33 (12.6)
	41–45	16 (6.1)
	46–50	4 (1.5)
	51–55	3 (1.1)
Years of working in the hospital	≤2 years	85 (32.6)
	3–5 years	42 (16.1)
	5–10 years	50 (19.2)
	11–15 years	46 (17.6)
	≥16 years	38 (14.6)
Education level	Junior college	5 (1.9)
	Bachelor’s degree	234 (89.7)
	Postgraduate	22 (8.4)
Department group	Internal medicine wards	114 (43.7)
	Surgical wards	127 (48.7)
	Other general inpatient wards	20 (7.7)
Professional title	Nurse	54 (20.7)
	Senior nurse	87 (33.3)
	Supervisor nurse	115 (44.1)
	Associate chief nurse or above	5 (1.9)
Average daily patient volume (unit)	≤30	7 (2.7)
	31–40	79 (30.3)
	41–50	156 (59.8)
	≥51	19 (7.3)
Monthly salary (CNY)	5,000–10,000	158 (60.5)
	10,001–15,000	83 (31.8)
	15,001–20,000	15 (5.7)
	≥20,000	5 (1.9)

Percentages may not sum to 100 due to rounding.

### Descriptive statistics and reliability

Descriptive statistics and internal consistency reliability for the main study variables are shown in Table 2.

**Table 2:** Descriptive statistics and reliability of study variables (*N* = 261).

Variable	Scoring	Min	Max	Mean	SD	Cronbach’s *α*
Nursing work interruptions (NWIS total)	Sum (12 items; 1–6)	14.00	72.00	40.14	10.97	0.889
Coping resources (total)	Sum (26 items; 1–5)	78.00	130.00	104.63	12.62	0.946
Burnout (CBI overall, 0–100)	Mean (19 items; 0–100)	0.00	100.00	41.89	21.22	0.962
Turnover intention	Mean (3 items; 1–5)	1.00	5.00	2.10	0.97	0.875

### Item-level and contextual patterns of nursing work interruptions

At the item level, the most frequently reported interruption sources were sudden patient requests (*M* = 5.31, SD = 1.03), sudden family member requests (*M* = 4.92, SD = 1.38), multiple simultaneous requests (*M* = 4.23, SD = 1.55), alarms from medical equipment or machines (*M* = 3.80, SD = 1.58), and new patient admissions or transfers (*M* = 3.74, SD = 1.46). These patterns suggest that the most common interruptions were mainly related to immediate interpersonal care demands and routine ward workflow events rather than rare emergency incidents.

However, the total NWIS scores did not differ significantly by department group. Mean scores were 40.45 (SD = 11.41) for internal medicine wards, 40.41 (SD = 10.77) for surgical wards, and 36.65 (SD = 9.46) for other general inpatient wards, *F*(2, 258) = 1.096, *p* = 0.336; Welch *F*(2, 55.981) = 1.416, *p* = 0.251. However, the total NWIS scores differed significantly by average daily patient volume, *F*(3, 257) = 3.945, *p* = 0.009; Welch *F*(3, 23.338) = 3.657, *p* = 0.027. Mean scores were 36.14 (SD = 11.80) for units with ≤30 patients per day, 37.37 (SD = 11.09) for 31–40 patients, 41.08 (SD = 10.61) for 41–50 patients, and 45.37 (SD = 10.57) for ≥51 patients. The Games–Howell *post-hoc* comparisons indicated that nurses in units with ≥51 patients per day reported significantly higher interruption scores than those in units with 31–40 patients per day (mean difference = 8.00, *p* = 0.032, 95% CI [0.56, 15.44]). No other pairwise comparisons were statistically significant.

### Correlations among main variables

The Pearson correlations among the four main variables are presented in Table 3.

**Table 3:** Pearson correlations among main variables (*N* = 261).

Variable	1	2	3	4
1. Interruptions (NWIS total)	1			
2. Coping resources (total)	−0.279^**^	1		
3. Burnout (CBI overall)	0.412^**^	−0.501^**^	1	
4. Turnover intention	0.168^**^	−0.268^**^	0.422^**^	1

^**^p < 0.01 (two-tailed) and NwisT–LizhiM *p* = 0.006.

### Mediation and serial mediation analyses (Models 4 and 6)

The results from mediation (Model 4) and serial mediation (Model 6) analyses are summarized in Tables 4A, 4B. Both unadjusted models and models adjusted for the department group, years of working in the hospital, and average daily patient volume are reported. In the adjusted models, the department group was entered using two dummy variables, with internal medicine wards as the reference group. Key path coefficients and bootstrapped indirect effects are provided.

**Table 4A:** Model 4 mediation results (*X* = NwisT; *M* = CbiM; and *Y* = LizhiM), *N* = 261.

Effect	Unadjusted *B*	*p*-value	95% CI/95% BC CI	Adjusted *B*^a^	*p*-value	95% CI 95% BC CI
Total effect (*c*): *X* → *Y*	0.0150	0.006	—	—	—	—
*a*-path: *X* → *M*	0.7961	<0.001	[0.5805, 1.0118]	0.7666	<0.001	[0.5471, 0.9862]
*b*-path: *M* → *Y* (controlling *X*)	0.0195	<0.001	[0.0139, 0.0251]	0.0187	<0.001	[0.0130, 0.0245]
Direct effect (*c’*): *X* → *Y* (controlling *M*)	−0.0006	0.917	[−0.0114, 0.0103]	−0.0001	0.990	[−0.0112, 0.0111]
Indirect effect (a*b): *X* → *M* → *Y*	0.0155	—	95% BC CI [0.0104, 0.0216]	0.0144	—	95% BC CI [0.0094, 0.0210]

a Adjusted models controlled for department group, years of working in the hospital, and average daily patient volume. The department group was entered using two dummy variables, with internal medicine wards as the reference group.

**Table 4B:** Model 6 mediation results (*X* = NwisT; *M*1 = Coping; *M*2 = CbiM; and *Y* = LizhiM), *N* = 261.

Effect	Unadjusted *B*	*p*-value	95% CI/95% BC CI	Adjusted *B*^a^	*p*-value	95% CI/95% BC CI
*a*1: *X* → *M*1	−0.3210	<0.001	[−0.4562, −0.1858]	−0.2981	<0.001	[−0.4347, −0.1615]
*a*2: *X* → *M*2 (controlling *M*1)	0.5704	<0.001	[0.3684, 0.7723]	0.5684	<0.001	[0.3610, 0.7757]
*d*21: *M*1 → *M*2 (controlling *X*)	−0.7034	<0.001	[−0.8790, −0.5279]	−0.6651	<0.001	[−0.8459, −0.4844]
*b*1: *M*1 → *Y* (controlling *X*, *M*2)	−0.0060	0.238	[−0.0159, 0.0040]	−0.0048	0.353	[−0.0149, 0.0053]
*b*2: *M*2 → *Y* (controlling *X*, *M*1)	0.0178	<0.001	[0.0116, 0.0241]	0.0175	<0.001	[0.0112, 0.0238]
Direct effect (*c*′): *X* → *Y* (controlling *M*1, *M*2)	−0.0012	0.832	[−0.0120, 0.0097]	−0.0005	0.923	[−0.0117, 0.0106]
Total indirect effect	0.0161	—	95% BC CI [0.0107, 0.0229]	0.0149	—	95% BC CI [0.0096, 0.0218]
Ind1: *X* → *M*1 → *Y*	0.0019	—	95% BC CI [−0.0022, 0.0065]	0.0014	—	95% BC CI [−0.0023, 0.0056]
Ind2: X → M2 → Y	0.0102	—	95% BC CI [0.0063, 0.0155]	0.0100	—	95% BC CI [0.0061, 0.0155]
Ind3: *X* → *M*1 → *M*2 → *Y*	0.0040	—	95% BC CI [0.0019, 0.0077]	0.0035	—	95% BC CI [0.0015, 0.0069]

a Adjusted models controlled for the department group, years of working in the hospital, and average daily patient volume. The Department group was entered using two dummy variables, with internal medicine wards as the reference group.

Unstandardized coefficients are reported. Indirect effects were estimated using 5,000 bias-corrected bootstrap samples (95% BC CIs); indirect effects were considered significant when CIs did not include zero.

#### Total effect (*c*)

In the regression model without mediators, interruptions were positively associated with turnover intention (*c* = 0.015; standard error, SE = 0.005; *t* = 2.746; *p* = 0.006).

Model 4 (Burnout as mediator; Table 4A).

Unadjusted Model 4: Burnout mediated the association between interruptions and turnover intention [indirect = 0.0155, 95% BC CI (0.0104, 0.0216)]. The direct effect was not significant (*c′* = −0.0006, *p* = 0.917).

Adjusted Model 4: After adjusting for the department group, years of working in the hospital, and average daily patient volume, the mediation remained significant. The department group was entered using two dummy variables, with internal medicine wards as the reference group. The indirect effect through burnout was 0.0144 [95% BC CI (0.0094, 0.0210)], and the direct effect remained non-significant (*c*′ = −0.0001, *p* = 0.990).

Model 6 (Serial mediation through coping resources and burnout, Table 4B).

Unadjusted Model 6: Indirect effects through burnout [0.0102, 95% BC CI (0.0063, 0.0155)] and serially through coping resources and burnout [0.0040, 95% BC CI (0.0019, 0.0077)] were significant. The coping-resources-only indirect effect was not significant [0.0019, 95% BC CI (−0.0022, 0.0065)]. The direct effect remained non-significant (c′ = −0.0012, *p* = 0.832).

Adjusted Model 6: The results remained robust after adjusting for department group, years of working in the hospital, and average daily patient volume. The department group was entered using two dummy variables, with internal medicine wards as the reference group. The burnout-only indirect effect was 0.0100 [95% BC CI (0.0061, 0.0155)], and serial indirect effect was 0.0035 [95% BC CI (0.0015, 0.0069)]. The coping-resources-only indirect effect remained non-significant [0.0014, 95% BC CI (−0.0023, 0.0056)]. The direct effect also remained non-significant (*c*′ = −0.0005, *p* = 0.923).

## Discussion

This study examined interruptions of nursing work as a psychosocial workplace exposure and tested a COR-informed mechanism linking interruptions to turnover intention through coping resources and burnout (4, 5, 23, 24). Three findings are notable. First, interruptions had a significant total association with turnover intention, but the direct effect became non-significant after including mediators. Second, burnout consistently emerged as the key mediator. The indirect effect through burnout was significant in both Model 4 and Model 6. Third, in the serial model, interruptions were associated with lower coping resources, higher burnout, and greater turnover intention through the proposed indirect pathway. Importantly, these observed associations remained robust after adjusting for the department group, years of working in the hospital, and average daily patient volume.

### Interpretation within a COR framework

The results are consistent with the core assumption of the COR theory, which proposes that stress and strain arise from threatened or depleted resources (4, 5). Within the context of inpatient nursing care, interruptions may repeatedly trigger attention shifts, reduce time for planned tasks, and undermine perceptions of control over the work environment (3, 25). This may be associated with depleted coping resources, the abilities and resources that help nurses cope with interruptions while maintaining task priorities (4, 5, 17). The present results suggest that lower coping resources were associated with higher burnout, which in turn was linked to withdrawal-related cognitions such as turnover intention (1, 2, 4, 5). The non-significant direct relationships in the mediation models also support that interruptions were associated with turnover intention through strained resources rather than directly influencing nurses’ turnover intentions.

These findings also align with recent international literature that emphasizes resilience and resource-related factors in the occupational stress and workforce outcomes of nurses. Evidence from pandemic and post-pandemic contexts suggests that stress and resilience remain important for nurses’ psychological adaptation and workforce stability (11, 12). Recent mediation and network studies further connect work engagement, resilience, burnout, and turnover intention among nurses (13, 14). Furthermore, the coping resources examined in the present study may be understood as work-related resources within a broader resilience-oriented occupational health framework. The present study extends this literature by focusing on nursing work interruptions as routine ward-level psychosocial exposures and by showing that their association with turnover intention was mainly linked to burnout, with coping resources positioned upstream in the proposed resource pathway.

### Coping resources as an upstream factor influencing burnout

Coping resources had a strong negative relationship with burnout, while interruptions were related to lower levels of coping resources. However, after controlling for burnout, there was no significant relationship between coping resources and turnover intention. This result is in accordance with theoretical predictions, as coping resources may be indirectly associated with turnover intention through burnout as a proximal predictor (1, 2, 4, 5). More precisely, strengthening coping resources is most relevant when they are associated with lower burnout, such as by increasing perceived control, improving coordination, and reducing unnecessary interruptions, rather than attempting to directly influence turnover intention through resource augmentation (4, 5).

### Occupational health and safety implications

The item-level findings further clarify the practical nature of nursing work interruptions in this sample. The most frequent interruptions were related to patient requests, family member requests, simultaneous demands from several people, equipment alarms, and admissions or transfers. These findings suggest that interruptions were embedded in routine ward workflow and interpersonal care demands, rather than being limited to rare emergency events. In addition, interruption scores did not differ significantly across department groups, but the scores were higher in units with the highest average daily patient volume compared with units caring for 31–40 patients per day. This pattern supports the relevance of workload-sensitive workflow management when designing interruption-reduction strategies.

From the perspective of occupational health and safety (OHS), interruptions are considered as a modifiable psychosocial work factor (3). While some interruptions are clinically important, others are avoidable or can be postponed. In this regard, interventions can be implemented on several levels, including the following (3, 25):

1. *Workflow and task design*: develop interruption-free zones or interruption-free times during tasks with high risk, such as the preparation and administration of medication, and design task sequences to reduce fragmentation (25).
2. *Communication norms and triage*: develop unit-level norms for urgent versus non-urgent communication, using structured communication systems to prevent interruptions during critical tasks (3).
3. *Staffing and workload management*: manage staffing levels and workload in relation to predictable peaks in interruption burden. Address the cumulative effects of resource depletion, including the need for adequate rest breaks (2, 4, 5, 26).
4. *Resource-supportive environments*: improve team-based support and supervisor responsiveness to help mitigate interruption-related overload. Ensure equitable scheduling and performance management to help mitigate chronic strain (2, 4, 5).

The observed mediation patterns remained similar after controlling for department group, years of working in the hospital, and average daily patient volume. These findings suggest that the associations were not limited to a single department group or workload category.

### Ethical salience without over-claiming moral distress

Although this research is based on an occupational health framework, the results support the notion that interruptions have ethical importance for nursing practice (27, 28). When interrupted care patterns limit continuous focus and time for patient-centered care, nurses may have difficulty fulfilling their professional and moral obligations (29, 30). From a COR theory perspective, nurses’ resources may be depleted and thus limit their ability to provide care that meets their professional standards (31). A key finding is that moral distress was not measured in this research. Thus, the implications are conceptual rather than empirical. However, they do support the role that working-environment conditions play in relation to ethical practice through patterns of resource depletion and burnout.

## Strengths

The study undertook a theory-driven modeling approach, which also assessed indirect effects through bias-corrected bootstrapping. This approach provided more informative mediation inferences than those obtained through statistical tests alone (22). Moreover, covariate-adjusted sensitivity analyses supported the robustness of the main indirect effects through burnout and the serial pathways, even after adjustment for the department group, years of working in the hospital, and average daily patient volume. The use of Chinese-language versions of well-established measures, along with high internal consistency reliability, enhanced the overall quality of the measures.

## Limitations

This study has several limitations. First, the cross-sectional design precludes conclusions about causality and temporal ordering. Although mediation models were used to examine theoretically informed indirect pathways, the findings should be interpreted as associations rather than evidence of cause-and-effect relationships. Longitudinal, diary-based, or intervention studies are needed to determine whether interruptions at work precede lower coping resources, burnout, and turnover intention over time. Second, since all main variables were measured using self-reported questionnaires at a single time point, common method variance may have inflated the observed associations. Although anonymity, standardized survey instructions, and the use of different validated scales were adopted to reduce this risk, common method variance cannot be completely ruled out. Future studies should incorporate multi-source data, objective indicators of interruptions or workload, and longitudinal or diary-based designs to reduce common method bias. Third, as the questionnaire was distributed through QR codes forwarded by head nurses, the exact number of nurses who received, viewed, or opened the survey link could not be verified; therefore, a formal response rate could not be calculated, and self-selection bias may have occurred. Fourth, although participants were recruited from 32 general inpatient wards across four campuses, all campuses belonged to the same tertiary hospital system and shared a unified nursing management structure, similar organizational policies, and broadly comparable ward workflows. Therefore, the findings may still reflect the organizational culture, staffing norms, communication practices, and workflow characteristics of the hospital system. In addition, campus identifiers were not collected; therefore, campus-specific differences could not be examined. The findings may not be directly generalizable to independent hospitals, lower-level hospitals, emergency departments, intensive care units, operating rooms, outpatient settings, or healthcare systems with different management structures. Fifth, subgroup analyses by the department group and average daily patient volume should be interpreted cautiously because some categories, particularly units with ≤30 patients per day and other general inpatient wards, had relatively small sample sizes.

## Future research

Future research needs to explore these mechanisms with longitudinal approaches. It also needs to test whether interventions aimed at reducing avoidable interruptions or increasing resource support can alleviate burnout and turnover intentions. It also remains crucial to distinguish types of interruption (such as necessary vs. avoidable or clinical vs. non-clinical) and whether certain resource areas (such as control and social support) are more effective in buffering burnout. Multi-site studies may help identify contextual factors that exacerbate or reduce interruption-related strain.

## Conclusion

Work interruptions were positively associated with turnover intention among ward nurses, and this relationship was primarily mediated by burnout. Serial mediation analyses further suggest that interruptions were associated with lower coping resources, higher burnout, and greater turnover intention within the proposed serial mediation pathway. Overall, these findings indicate that interruptions were associated with turnover intention mainly through burnout, with coping resources functioning as an upstream factor in the resource-depletion pathway. These results highlight interruptions as a modifiable occupational health factor and underscore the relevance of organizational strategies aimed at reducing avoidable disruptions and strengthening resource-supportive environments. Such measures may protect nurse wellbeing and stabilize the workforce.

## Data Availability

The original contributions presented in the study are included in the article/supplementary material, further inquiries can be directed to the corresponding author.
